# Neutrophil extracellular traps induced by *Haemonchus contortus* excretory–secretory proteins varies among goats, gerbils, and mice

**DOI:** 10.1186/s13071-025-06956-z

**Published:** 2025-07-28

**Authors:** Yangchun Tan, Shuyi Cao, Muhammad Azhar Memon, Zhaohai Wen, Cheng Chen, Jiajun Feng, Xiaokai Song, Lixin Xu, Mingmin Lu, Ruofeng Yan

**Affiliations:** MOE Joint International Research Laboratory of Animal Health and Food Safety, College of Veterinary Medicine, Nanjing Agricultural University, Nanjing, Jiangsu 210095 People’s Republic of China

**Keywords:** Neutrophils, *Haemonchus contortus*, NETs, HcESPs

## Abstract

**Background:**

Previous studies indicated that infection with *Haemonchus contortus* is host-specific (goat: susceptible host; gerbil: paratenic host; mouse: resistant host). Neutrophils play an essential role in host defense against parasitic infection through phagocytic engulfment, reactive oxygen species (ROS) generation, and neutrophil extracellular traps (NETs) formation. NETs are large web-like complexes consisting of a DNA scaffold decorated with various proteins components, including histones, myeloperoxidase, and elastase. They are released through both ROS-dependent and ROS-independent pathways. Previous studies have demonstrated both constraints and effectiveness of NETs in helminths. However, the roles of NETs in anti-infection of *H. contortus* in different hosts are still unclear.

**Methods:**

To assess host-specific variations in NETs release, neutrophils isolated from goats, gerbils, and mice were co-cultured with *Haemonchus contortus* third-stage larvae (HcL3), followed by quantitative analysis of NETs formation using the PicoGreen® fluorescence assay. Subsequently, *H. contortus* excretory–secretory proteins (HcESPs) were co-cultured with neutrophils isolated from each host species. NETs release and ROS production were then quantitatively assessed using PicoGreen® fluorescence intensity and oxidation-sensitive dichlorodihydrofluorescein diacetate (DCFH-DA) fluorescence. In addition, the neutrophil’s phagocytic ability for FITC-dextran was evaluated by flow cytometric analysis. Finally, to elucidate the signaling pathways involved in HcESP-induced NETs release in goat neutrophils, four specific inhibitors were employed for pretreatment prior to stimulation.

**Results:**

Our results demonstrate that in vitro stimulation with HcL3 triggers NETs formation. The release of NETs exhibits significant host-specific variation, specifically, neutrophils from mice showed the highest NETs release, followed by gerbils, and a minimal response in goats. Moreover, HcESP treatment markedly inhibited ROS generation and phagocytic capacity in neutrophils from all three host species. Intriguingly, HcESPs exerted host-specific modulation of NETs release, with inhibition observed in goats, enhancement in mice, and context-dependent modulation in gerbils. Mechanistic investigations revealed that the NETs suppression in goats neutrophils involved both nicotinamide adenine dinucleotide phosphate (NADPH) oxidase- and neutrophil elastase-dependent pathways.

**Conclusions:**

Our results demonstrate that HcESPs significantly inhibit NETs formation in goat neutrophils through dual modulation of NADPH oxidase and neutrophil elastase activity. This finding highlights these two enzymes as promising molecular targets for anti-helminthic vaccine development.

**Graphical abstract:**

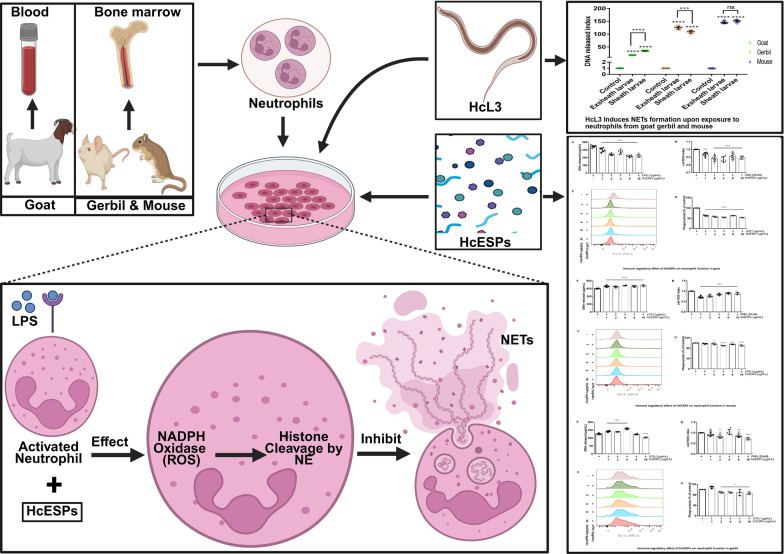

**Supplementary Information:**

The online version contains supplementary material available at 10.1186/s13071-025-06956-z.

## Background

*Haemonchus contortus* is a hematophagous gastrointestinal nematode that colonizes the abomasum of small ruminants, causing significant blood loss. This parasite causes substantial economic losses on the global livestock industry. The rapid spread of anthelmintic-resistant in *H. contortus* populations has intensified the need for alternative control measures. Among these, immunological interventions targeting critical host–parasite interfaces represent a particularly promising approach for sustainable management of haemonchosis [1, 2]. During infection, *H. contortus* evades host immune defenses through secretion of complex excretory–secretory products (ESPs), comprising proteins, peptides, nucleic acids, lipids, polysaccharides, and small organic molecules [3]. Such immunomodulatory ESPs have been characterized across diverse helminths, including trematodes, cestodes, and nematodes, emerging as promising diagnostic and therapeutic targets [4, 5]. However, the precise molecular mechanisms mediating these immunomodulatory effects require further elucidation.

As key effectors of innate immunity, neutrophils (polymorphonuclear leukocytes, PMN) constitute the primary defense against bacterial [6, 7], fungal [8, 9], viral [10], and parasitic [11] pathogens through phagocytosis, ROS production, and neutrophil extracellular traps (NETs) formation [12]. NETs—extracellular DNA scaffolds decorated with antimicrobial proteins—represent a crucial antimicrobial mechanism that limits pathogen dissemination [13]. Recent advances in neutrophil biology have highlighted NETs immunoregulatory functions [14], while pathogens have developed sophisticated evasion tactics, such as NETs inhibition, nuclease-mediated degradation, and surface modification to evade entrapment [15, 16]. Parasites demonstrate remarkable diversity in their induction of neutrophil extracellular trap formation (NETosis): *Leishmania amazonensis* promastigotes trigger classical ROS-dependent NETs formation [17], whereas *Entamoeba histolytica* employs noncanonical pathways [18]. Notably, some helminths leverage their symbiotic microbiota to directly stimulate NETs release. Despite these findings, the functional relevance of NETs in parasitic infections remains insufficiently understood, highlighting the need for comprehensive studies to elucidate their mechanistic contributions to host–parasite interactions.

Goats serve as susceptible hosts for *Haemonchus contortus*, typically developing characteristic clinical manifestations including severe anemia and hypoalbuminemia upon infection and parasite establishment [19]. In contrast to goat hosts, *H. contortus* third-stage larvae (HcL3) demonstrate delayed growth and developmental arrest in gerbils, failing to attain sexual maturity [20]. This biological evidence confirms gerbils as paratenic hosts for *H. contortus* [21, 22]. Mice, as resistant hosts, show markedly reduced susceptibility to *H. contortus* infection. Neutrophils, as key effector cells of innate immunity, are rapidly recruited to infection sites upon HcL3 invasion. Therefore, examining the relationship between neutrophil-mediated immune responses and host resistance to nematodes presents a crucial research direction [23]. Elucidation of these mechanisms will not only enhance our knowledge of host-parasite immunobiology but may also facilitate the development of innovative anti-helminthic vaccines and therapeutic agents.

## Methods

### Parasites and animals

The Nanjing strain of *Haemonchus contortus* was maintained in helminth-free goats at the Laboratory of Veterinary Parasitology, Nanjing Agricultural University, China, following established protocols [24]. Eggs were cultured and HcL3 were harvested using previously described methods [25, 26].

For experimental animals, healthy local goats (5–7 months old) were housed in ventilated cages under nematode-free conditions with ad libitum access to alfalfa pellets, hay, and water. Gerbils (70–100 g) were obtained from the Zhejiang Experimental Animal Center (China), and 25–30 g BALB/c mice were sourced from the Jiangsu Experimental Animal Center (China). All animals were maintained under sterile conditions in the Animal Experimental Laboratory of Nanjing Agricultural University.

### Collection of HcESPs

Following euthanasia of a single infected goat, adult *Haemonchus contortus* specimens were immediately collected from the abomasal lumen using established techniques [27]. HcESPs were subsequently isolated following previously published protocols [28].

### Isolation of neutrophils

Neutrophils were isolated from mouse and gerbil bone marrow, as well as goat peripheral blood, using Percoll density gradient centrifugation. After two washes with sterile phosphate-buffered saline (PBS), Wright–Giemsa stain was used for assessing cellular purity.

### Culture of neutrophils with HcL3

Neutrophils (1 × 10^6^ cells/well) were co-cultured with 100 HcL3 larvae in poly-l-lysine hydrobromide-coated (Acmec Biochemical, Shanghai, China) round coverslips and incubated in a CO_2_ incubator for 3 h.

### Detection of NETs by immunofluorescence assay

After overnight fixation in 4% paraformaldehyde at 4°C, neutrophils and larvae were permeabilized (PBS with 1% fetal bovine serum [FBS] and 0.05% Triton X-100, 24 h) then blocked with 5% bovine serum albumin (BSA; 1 h, room temperature [RT]). Samples were then incubated with primary antibodies (mouse anti-myeloperoxidase monoclonal antibody) at 4 °C overnight, followed by Cy3-conjugated goat anti-mouse IgG secondary antibody (Shenggong Bioengineering, Shanghai, China) for 1 h. After thorough washing with PBST, nuclei were counterstained with Hoechst 33342 (1 μg/mL) for 5 min. Slides were mounted with antifade medium and imaged using an EVOS FL Auto inverted fluorescence microscope.

### Quantification of DNA released from neutrophils

After 3 h co-culture of neutrophils with HcL3 or HcESPs, supernatants were collected and centrifuged (500 × *g*, 5 min) to obtain cell-free fractions. Extracellular DNA content was quantified using the PicoGreen dsDNA Quantitation Assay Kit according to the manufacturer’s protocol. Samples were assayed in 96-well plates using a microplate reader (EnSpire, PerkinElmer, USA).

### Effect of HcESPs on ROS release

Neutrophils were randomly allocated into experimental and control groups (*n* = 3 replicates per group). Cells (1 × 10^6^/well) were loaded with 10 μM DCFH-DA at 37 °C for 20 min with intermittent gentle agitation (every 3–5 min). Following three PBS washes to remove unincorporated probe, all groups were stimulated with 25 nM phorbol 12-myristate 13-acetate (PMA) for 1 h. Experimental groups received graded concentrations of HcESPs (0, 1, 2, 4, 8, 16 μg/mL), while control groups received vehicle alone. Samples were incubated at 37 ℃ for 1 h. Intracellular ROS production was assayed in 96-well plates using a microplate reader.

### Effect of HcESPs on phagocytic function

Neutrophils were pretreated with 1 μg/mL lipopolysaccharide (LPS; Acmec Biochemical, Shanghai, China), then incubated with HcESPs (0, 1, 2, 4, 8, 16 μg/mL) for 3 h under standard conditions. Following two PBS washes, cells were resuspended in 100 μL PBS and incubated with 14 μM FITC-dextra for 1 h at 37 °C. After additional PBS washes, samples were analyzed by flow cytometry (CytoFLEX, Beckman, USA).

### Effect of inhibitors on NETs pathway

Neutrophils (1 × 10^6^ cells/well) were resuspended in complete RPMI 1640 medium and pre-incubated at 37 °C for 30 min. After two PBS washes, cells were treated for 30 min with either vehicle control (PBS) or one of four specific inhibitors, including elastase inhibitor III (NEi; 10 μM), myeloperoxidase inhibitor-I (MPOi; 5 μM), GSK484 hydrochloride (peptidylarginine deiminase 4 inhibitor, PAD4i; 50 μM), and diphenyleneiodonium chloride (DPI; 1 μM). Following additional PBS washes, cells were stimulated with 1 μg/mL LPS for 1 h before HcESP treatment (0–16 μg/mL). After 3 h incubation, culture supernatants were collected and mixed 1:1 with PicoGreen dsDNA quantitation reagent (100 μL each) in 96-well plates. Samples were assayed in 96-well plates using a microplate reader.

### Statistical analysis

Statistical analyses were performed using IBM SPSS Statistics 25 (IBM, USA), with *t*-tests and one-way analysis of variance (ANOVA) for group comparisons. Graphical representations were generated using GraphPad Prism 7.0 (GraphPad Prism, USA). Data are presented as the mean ± standard deviation, with statistical significance denoted as *P < 0.05, **P < 0.01, ***P < 0.001, and ***P < 0.0001. All experiments were repeated a minimum of three times. Flow cytometry data were analyzed using FlowJo software (version 10, USA).

## Results

### NETs release from neutrophils differentially among host species

Neutrophils isolated from goats, gerbils, and mice exhibited > 95.0% purity and preserved immunocompetence, as confirmed by functional assays (Additional file: Fig. S1A, B).

To assess NETs formation, neutrophils were incubated with live or heat-inactivated HcL3 (100 ℃, 10 min) for 3 h and analyzed by immunofluorescence microscopy. Live HcL3 triggered robust NETs release, visualized as extracellular DNA filaments colocalized with myeloperoxidase (Additional file: Fig. S2B, C). In contrast, heat-inactivated HcL3 failed to induce NETosis (Additional file: Fig. S2A).

**Fig. 1:**
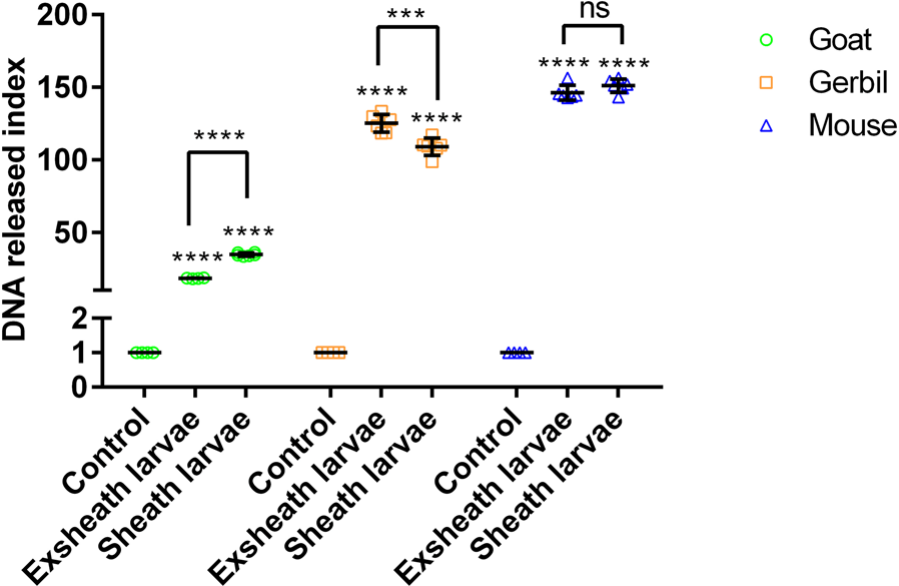
HcL3 induces NETs formation upon exposure to neutrophils from goats, gerbils, and mice for 3 h. The neutrophil-only group served as the control. The DNA released index was determined by setting the control group as 1. HcL3, *Haemonchus contortus* third-stage larvae, Exsheath larvae, exsheath HcL3, Sheath larvae, sheath HcL3, NETs, neutrophil extracellular traps

Quantitative extracellular DNA analysis revealed that exsheathed and sheathed HcL3 significantly induced NETs release in goats (exsheath: *t*-test, *t*_(10)_ =  −219.942, *P* < 0.0001; sheath: *t*-test, *t*_(10)_ =  −72.193, *P* < 0.0001), gerbils (exsheath: *t*-test, *t*_(10)_ =  −50.019, *P* < 0.0001; sheath: *t*-test, *t*_(10)_ =  −44.312, *P* < 0.0001), and mice (exsheath: *t-*test, *t*_(10)_ =  −69.247, *P* < 0.0001; sheath: *t*-test, *t*_(10)_ =  −80.385, *P* < 0.0001) (Fig. 1). Among the three hosts, mice exhibited the highest NETs release, followed by gerbils, whereas goats exhibited the lowest. Comparative approaches revealed that sheathed larvae significantly induced NETs release more than exsheathed larvae in goats (*t*-test, *t*_(10)_ =  −34.552, *P* < 0.0001). In gerbils, exsheathed larvae induced NETs release more than sheathed (*t*-test, *t*_(10)_ = 4.67, *P* = 0.001), while there is no significant difference in mice (*t*-test, *t*_(10)_ =  −1.742, *P* = 0.112).

### HcESPs differentially regulate NETs release across host species

**Fig. 2:**
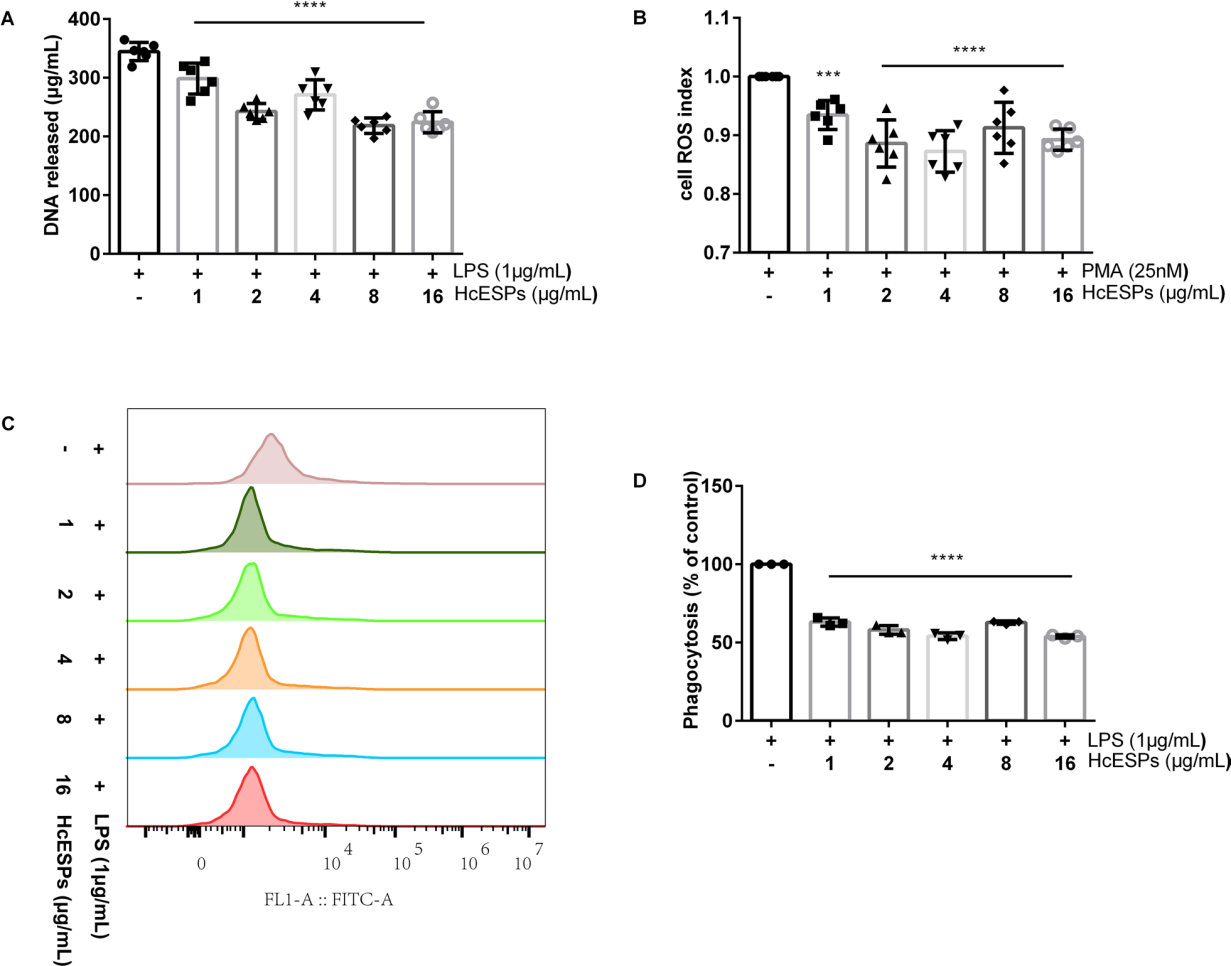
Modulation of neutrophil function by HcESPs in goats. **A** Effect of HcESPs on NETs formation in goats. **B** Effect of HcESPs on ROS production in goat neutrophils.** C** Assessment of neutrophil phagocytic function in goats using flow cytometry. **D** Phagocytic activity of goat neutrophils was quantified using flow cytometry and analyzed with FlowJo software (Version 10, USA). PMA, phorbol 12-myristate 13-acetate, LPS, lipopolysaccharide, HcESPs, *Haemonchus contortus* excretory–secretory proteins, ROS, reactive oxygen species

HcESPs are composed of complex biomolecular mixtures with molecular weights ranging from 10 to 180 kDa (Additional file: Fig. S3). Compared with the control group without HcESPs, increasing HcESP concentrations exhibited inhibitory effects on NETs release (1–16 μg/mL: ANOVA, *F*_(5,30)_ = 37.261, *P* < 0.0001; Fig. 2A), ROS production (1 μg/mL: ANOVA, *F*_(5,30)_ = 13.605, *P* = 0.001; 2–16 μg/mL: ANOVA, *F*_(5,30)_ = 13.605, *P* < 0.0001; Fig. 2B), and the phagocytic function (1–16 μg/mL: ANOVA, *F*_(5,12)_ = 258.596, *P* < 0.0001; Fig. 2C, D) of goat neutrophils. The suppression of goat NETs release by HcESPs is ROS-dependent.

**Fig. 3:**
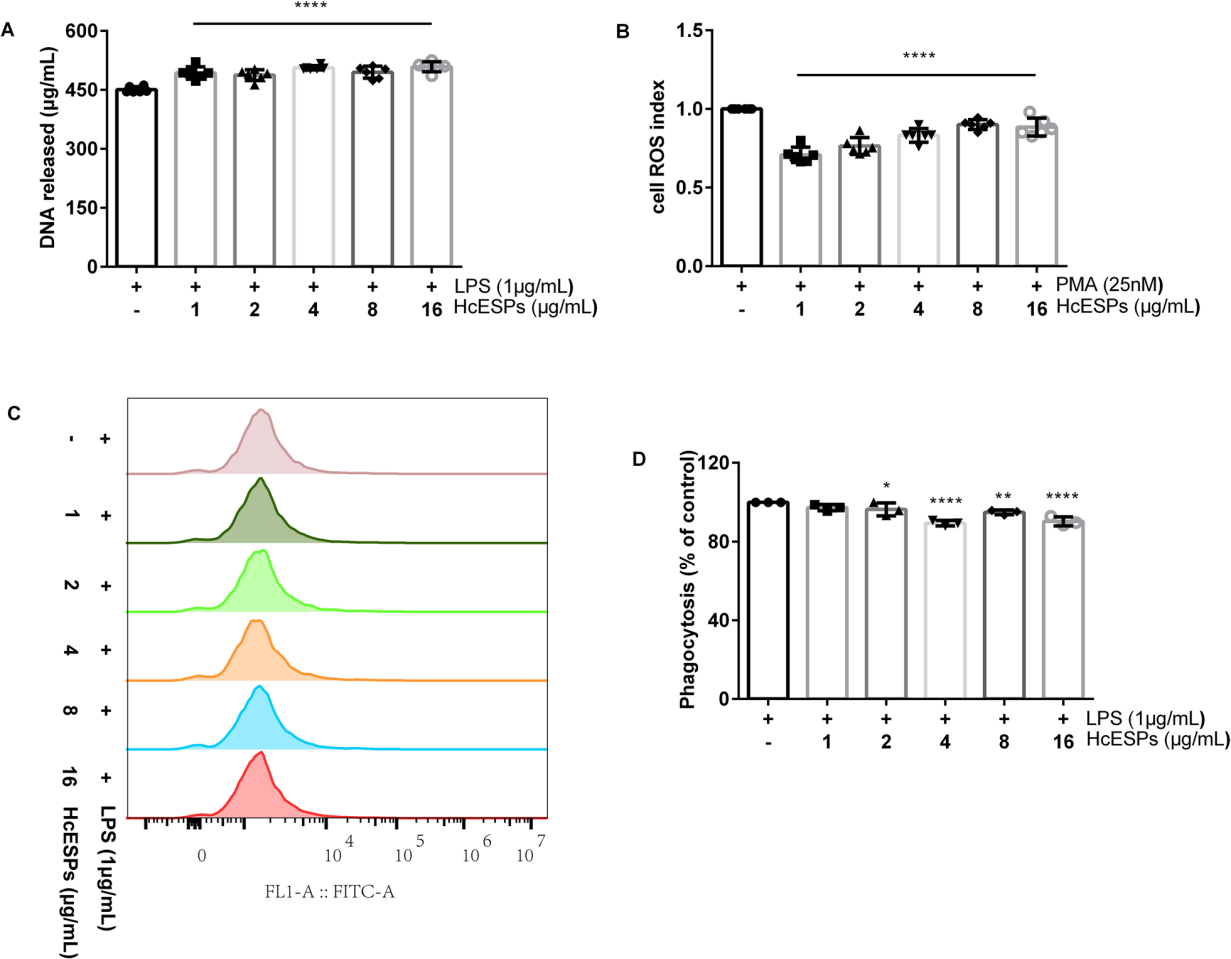
Modulation of neutrophil function by HcESPs in mice. **A** Effect of HcESPs on NETs formation in mice. **B** Effect of HcESPs on ROS production in mouse neutrophils. **C** Assessment of neutrophil phagocytic function in mouse using flow cytometry. **D** Phagocytic activity of mouse neutrophils was quantified using flow cytometry and analyzed with FlowJo software (Version 10, USA). PMA, phorbol 12-myristate 13-acetate, LPS, lipopolysaccharide, HcESPs, *Haemonchus contortus* excretory-secretory proteins, ROS, reactive oxygen species

In mice, higher doses of HcESPs significantly enhanced NETs formation (1–16 μg/mL: ANOVA, *F*_(5,30)_ = 17.212, *P* < 0.0001; Fig. 3A) while simultaneously suppressing ROS production (1–16 μg/mL: ANOVA, *F*_(5,30)_ = 33.98, *P* < 0.0001; Fig. 3B). The enhancement of NETs formation in mice appears to be regulated through a ROS-independent mechanism. HcESPs also suppressed neutrophil phagocytic function (1 μg/mL: ANOVA, *F*_(5,12)_ = 13.64, *P* = 0.113; 2 μg/mL: ANOVA, *F*_(5,12)_ = 13.64, *P* = 0.046; 4 μg/mL: ANOVA, *F*_(5,12)_ = 13.64, *P* < 0.0001; 8 μg/mL: ANOVA, *F*_(5,12)_ = 13.64, *P* = 0.008; 16 μg/mL: ANOVA, *F*_(5,12)_ = 13.64, *P* < 0.0001; Fig. 3C, D).

**Fig. 4:**
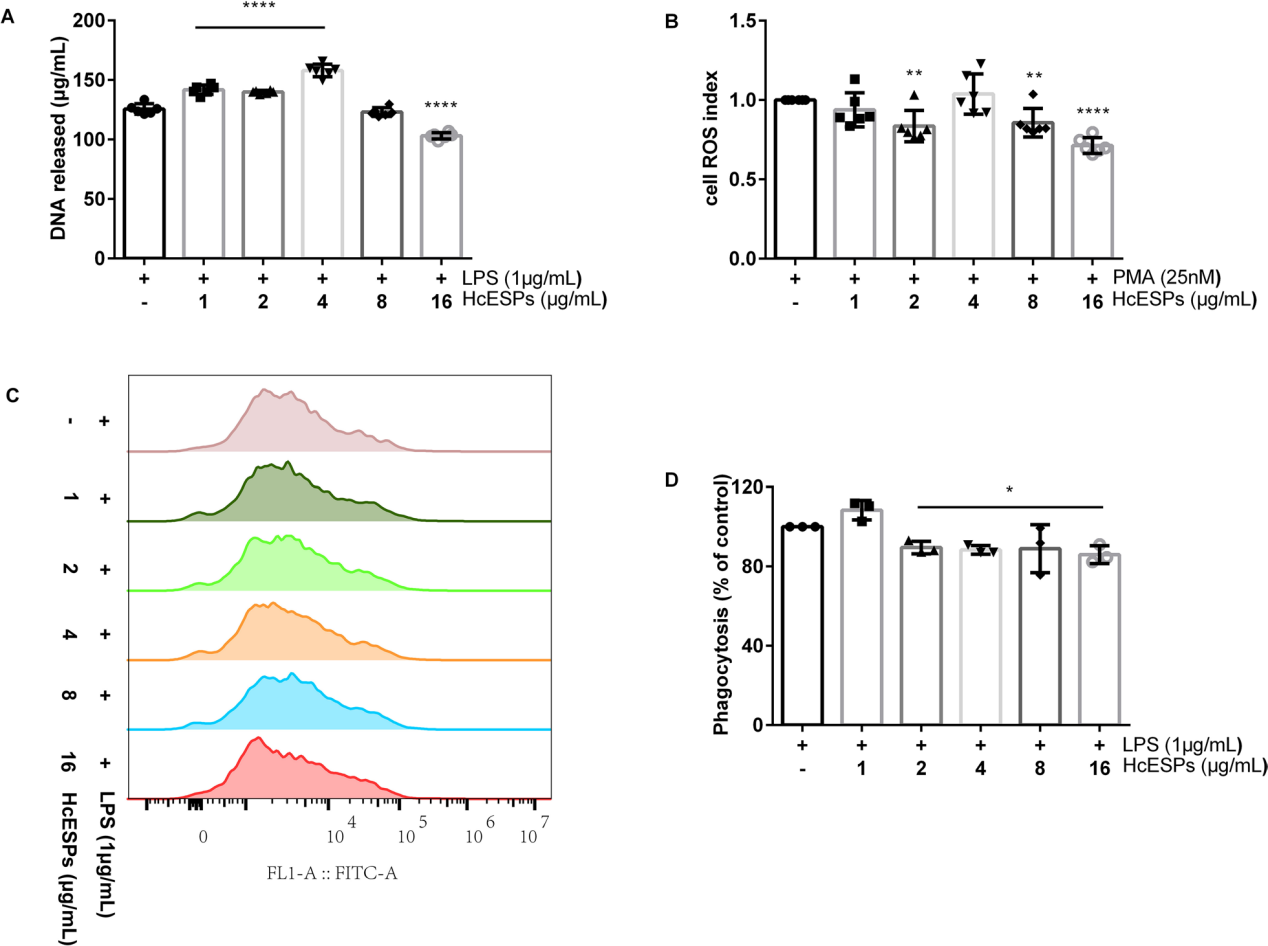
Modulation of neutrophil function by HcESPs in gerbils. **A** Effect of HcESPs on NETs formation in gerbils. **B** Effect of HcESPs on ROS production in gerbil neutrophils.** C** Assessment of neutrophil phagocytic function in gerbils using flow cytometry. **D** Phagocytic activity of gerbil neutrophils was quantified using flow cytometry and analyzed with FlowJo software (Version 10, USA). PMA, phorbol 12-myristate 13-acetate, LPS, lipopolysaccharide, HcESPs, *Haemonchus contortus* excretory-secretory proteins, ROS, reactive oxygen species

In gerbils, HcESPs at concentrations of 1, 2, and 4 μg/mL significantly increased NETs formation compared with the control group. However, at a concentration of 16 μg/ml, HcESPs led to a significant reduction in NETs formation (1 μg/mL: ANOVA, *F*_(5,30)_ = 133.335, *P* < 0.0001; 2 μg/mL: ANOVA, *F*_(5,30)_ = 133.335, *P* < 0.0001; 4 μg/mL: ANOVA, *F*_(5,30)_ = 133.335, *P* < 0.0001; 8 μg/mL: ANOVA, *F*_(5,30)_ = 133.335, *P* = 0.272; 16 μg/mL: ANOVA, *F*_(5,30)_ = 133.335, *P* < 0.0001; Fig. 4A). ROS production was suppressed at HcESP concentrations of 2, 8, and 16 μg/ml (1 μg/mL: ANOVA, *F*_(5,30)_ = 10.741, *P* = 0.24; 2 μg/mL: ANOVA, *F*_(5,30)_ = 10.741, *P* = 0.003; 4 μg/mL: ANOVA, *F*_(5,30)_ = 10.741, *P* = 0.468; 8 μg/mL: ANOVA, *F*_(5,30)_ = 10.741, *P* = 0.009; 16 μg/mL: ANOVA, *F*_(5,30)_ = 10.741, *P* < 0.0001; Fig. 4B), suggesting that the effect of HcESPs on NETs formation in gerbils occurs via a ROS-independent mechanism. Furthermore, HcESPs simultaneously suppressed neutrophil phagocytic activity (1 μg/mL: ANOVA, *F*_(5,12)_ = 6.704, *P* = 0.106; 2 μg/mL: ANOVA, *F*_(5,12)_ = 6.704, *P* = 0.049; 4 μg/mL: ANOVA, *F*_(5,12)_ = 6.704, *P* = 0.031; 8 μg/mL: ANOVA, *F*_(5,12)_ = 6.704, *P* = 0.039; 16 μg/mL: ANOVA, *F*_(5,12)_ = 6.704, *P* = 0.012; Fig. 4C, D).

### HcESPs-mediated NETosis depends on NADPH oxidase and neutrophil elastase (NE)

**Fig. 5:**
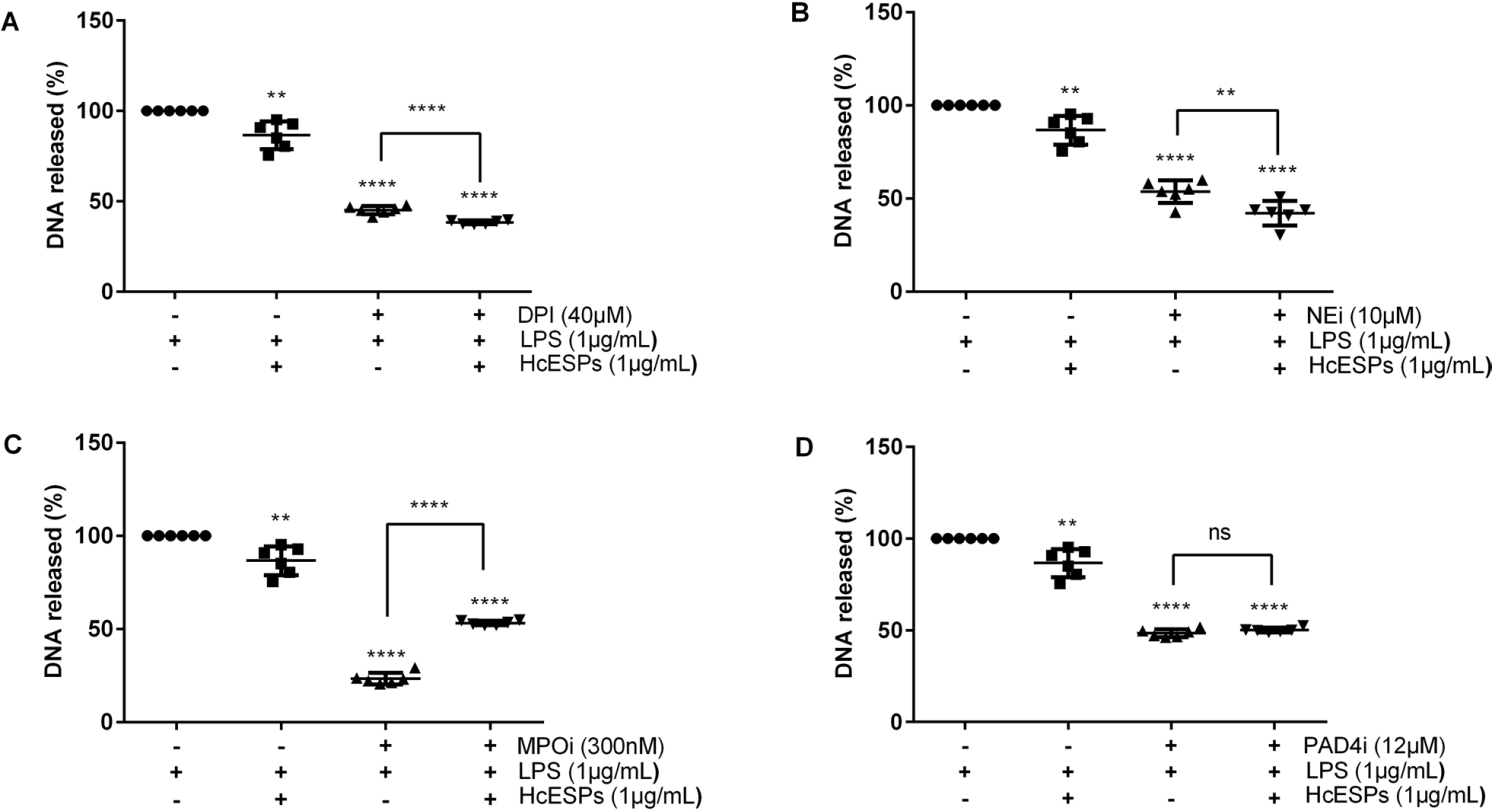
HcESPs inhibit goats NETs formation via NADPH oxidase and NE-dependent mechanisms. Neutrophils were pretreated with or without inhibitors for 30 min, stimulated with 1 μg/mL LPS (1 h), then exposed to 0 or 1 μg/mL HcESPs (3 h). **A** DPI significantly enhance HcESP-mediated suppression of NETosis. **B** NEi enhance NETs inhibition by HcESPs. **C** MPOi reversed NETs suppression, enhancing DNA release.** D** PAD4i had no significant effect on HcESP activity. NE, neutrophil elastase, LPS, lipopolysaccharide, DPI, diphenyleneiodonium chloride, NETosis, neutrophil extracellular trap formation, NEi, neutrophil elastase inhibitor, MPOi, myeloperoxidase inhibitor, PAD4i, peptidylarginine deiminase 4 inhibitor

Compared with the control (no inhibitors and HcESPS), HcESPs significantly suppressed NETosis (*t*-test, *t*_(10)_ = 4.276, *P* = 0.008). This suppression was further enhanced by pretreatment with either DPI or NEi (DPI: *t*-test, *t*_(10)_ = 6.494, *P* < 0.0001; NEi: *t*-test, *t*_(10)_ = 3.184, *P* = 0.01) (Fig. 5A, B), confirming the involvement of both enzymes in HcESP-mediated NETosis inhibition. In contrast, pretreatment with MPOi resulted in a marked increase in NETs release (*t*-test, *t*_(10)_ = − 22.026, *P* < 0.0001) (Fig. 5C), suggesting that myeloperoxidase may play role in the regulatory pathway. However, PAD4 inhibition had no significant impact on NETs formation (*t*-test, *t*_(10)_ = −1.691, *P* = 0.122) (Fig. 5D), implying that PAD4 is not essential for the NETs response under these conditions.

## Discussion

During protozoan infections, neutrophils exert their antimicrobial effects primarily through phagocytosis, ROS production, and other cytotoxic mechanisms. These effector functions have been extensively characterized in controlling infections by *Plasmodium* [29, 30], *Leishmania* [31], and *Trypanosoma cruzi* through phagocytosis and ROS production [32]. Neutrophils can also disrupt membrane integrity of pathogens such as *Trichomonas vaginalis* through phagocytic activity [33]. In contrast, anti-helminth defenses face unique challenges due to the parasites’ macroscopic size, which precludes conventional phagocytosis. Early studies demonstrated that neutrophils promote helminth degradation by disrupting cuticular layers [34, 35], facilitated by specific binding to surface-bound IgM and IgG that target epidermal crypt structures [36]. These interactions induce characteristic pathological changes including cuticular swelling, focal collapse, and tissue lysis [37]. The discovery of NETs has expanded our understanding of anti-helminth immunity. NETs contribute to parasite control during primary *Nippostrongylus brasiliensis* infection [38], while in secondary infections, N2-polarized neutrophils enhance macrophage differentiation into M2 phenotypes via interleukin (IL)-13 secretion, accelerating parasite clearance [39, 40]. Complement-mediated synergy between neutrophils and macrophages further facilitates *Strongyloides stercoralis* elimination through extracellular trap mechanisms [41, 42]. The role of neutrophils in *H. contortus* infection remains not fully elucidated, where bovine neutrophils exhibit three distinct morphological patterns of NETosis (diffuse, aggregated, and spread) to immobilize HcL3 [43]. These NETs structures effectively limit larval migration to infection sites, thereby modulating disease pathogenesis. Furthermore, in the adaptive immune response, HcL3 stimulated neutrophils rapidly secrete IL-4, initiating a Th2-polarized immune cascade that enhances parasite clearance [44]. Neutrophils are established to be important in determining host susceptibility to HcL3 larvae, where comparative studies demonstrate resistant St. Croix hair lambs (STC) mount a robust neutrophil response featuring rapid peripheral neutrophilia, significant rumen mucosal infiltration, and strong IgA production that collectively delay HcL3 development into fourth-stage larvae (HcL4); promoting parasite expulsion while reducing adult worm burdens and shortening infection duration and severity [45, 46]. Among them, in vitro studies reveal HcL3 potently induce NETosis in resistant and susceptible lambs. Notably, neutrophils from resistant lambs exhibit higher HcL3-binding affinity than those from susceptible lambs [47]. This is consistent with our results that HcL3 can induce the release of NETs in goats, gerbils, and mice. The higher number of NETs released by neutrophils in resistant mice, compared with susceptible goats, may be attributed to differences in the binding affinity between neutrophils and HcL3. Therefore, the influence of neutrophil–parasite binding affinity on anti-helminth mechanisms warrants further investigation.

Parasite–host interactions involve complex immunological dynamics, where hosts employ multifaceted defense mechanisms, while parasites evolve sophisticated immune evasion strategies [48]. During early parasitic infections, host neutrophils exert antiparasitic effects primarily through NETs release [49]. However, parasites have evolved sophisticated strategies to evade this defense mechanism by secreting immunomodulatory molecules, including *Trypanosoma evansi*, which secretes TatD DNases; *Leishmania* parasites, which express 3′-nucleotidase/nuclease (3′NT/NU) that degrade NETs [50, 51]; *Trichinella spiralis*, which produces secreted serine protease inhibitors that disrupt NETs-associated antimicrobial proteins [52]; *Trichinella spiralis*, with excretory–secretory (ES) antigens that inhibit the release of NETs [53]; and *Mesochondrides cortex* excretory/secretory factors that inhibit ROS-induced NETs by blocking the activation of the nonselective calcium permeable channel transient receptor potential melastatin 2 [54]. While previous studies have established the role of HcESPs in modulating adaptive immunity [55, 56], their function in innate immune regulation remains poorly characterized [57]. As HcESPs comprise a complex mixture of proteins and glycoproteins that may independently or synergistically modulate NETosis through distinct pathways, our in vitro findings specifically demonstrate their potential to facilitate HcL3 infection by suppressing NETs formation. Characterization of specific NETs-inhibitory components within HcESPs will establish a theoretical foundation for developing targeted anthelmintics and vaccines, representing a key direction for our future investigations.

NETs represent intricate macromolecular complexes composed of dsDNA scaffolds associated with antimicrobial proteins such as histones, myeloperoxidase (MPO), neutrophil elastase (NE), defensins, and cationic antimicrobial peptides [58]. These structures are released through a specialized cell death process called NETosis [59], which proceeds through two main mechanisms—ROS-dependent pathways that can be further classified as either NADPH oxidase (NOX)-dependent or NOX-independent, and ROS-independent pathways. Within the NOX-dependent pathway, both MPO and NOX serve as critical regulators [60, 61], where MPO works in concert with serine proteases and NE to facilitate chromatin decondensation via histone cleavage [62]. This process is further enhanced through MPO’s interaction with DEK (protein encoded by proto-oncogene *dek*) and protein-arginine deiminase 4 (PAD4)-mediated histone citrullination, which promotes chromatin depolymerization [63]. Our experimental results demonstrate that HcESPs suppress NETosis via a ROS-dependent mechanism. Pretreatment of neutrophils with DPI, a specific NOX inhibitor, significantly reduced NETs release in HcESP-treated groups compared with controls, confirming that HcESPs inhibit NETosis in caprine neutrophils through a NOX-dependent pathway regulated by downstream NE. NETs play significant roles in parasitic infections and associated pathologies. Previous studies indicate that histone antibodies can block NETs-mediated killing of *Leishmania amazonensis* [64], while NETs quantification serves as a biomarker for disease progression and treatment efficacy in cutaneous leishmaniasis and malaria [65, 66]. Furthermore, recombinant human DNase or elastase inhibitors have been shown to mitigate *Plasmodium berghei*-induced pulmonary pathology and improve murine survival [67]. The NOX-dependent ROS production and subsequent NETs release constitute crucial antimicrobial defenses. Targeting NADPH oxidase as a vaccine component may enhance pathogen clearance, immune regulation to prevent excessive inflammation, and host defense mechanisms [68–71]. Although NE is essential for pathogen clearance, its dysregulated activity contributes to tissue damage in emphysema, cystic fibrosis, and chronic obstructive pulmonary disease (COPD) [72–74]. Therapeutic modulation of elastase activity could therefore alleviate chronic inflammatory tissue injury [75]. These findings suggest that investigating NOX and NE pathways in parasitic infections may provide foundational insights for developing novel vaccines or adjuvants. 

## Conclusions

Variation in host susceptibility to *Haemonchus contortus* infection may relate to NETosis responses. We provide preliminary evidence that HcESPs impair goat neutrophil function through dual inhibition of NE and NOX-dependent NETosis pathways. However, specific immunomodulatory components from HcESPs and their precise molecular targets remain unknown, this represents a key direction for our future investigations.

## Supplementary Information

Supplementary Material 1.

## Data Availability

Data supporting the main conclusions of this study are included in the manuscript.

## Abbreviations

*H. contortus*: *Haemonchus contortus*
HcL3: *H. contortus* third-stage larvae
NETs: Neutrophil extracellular traps
HcESPs: *H. contortus* excretory–secretory protein
ROS: Reactive oxygen species
FBS: Fetal bovine serum
LPS: Lipopolysaccharide
PMN: Polymorphonuclear leukocyte
PMA: Phorbol 12-myristate 13-acetate
MPO: Myoperoxidase
*Nb*: *Nippostrongylus brasiliensis*
NADPH: Nicotinamide adenine dinucleotide phosphate
DPI: Diphenyleneiodonium chloride
NE: Neutrophil elastase
PBS: Phosphate-buffered saline
