# Supplementary material for: Neutrophil extracellular traps induced by Haemonchus contortus excretory–secretory proteins varies among goats, gerbils, and mice
Source: Parasit Vectors. 2025 Jul 28;18:304. doi: 10.1186/s13071-025-06956-z (PMC12302446; doi:10.1186/s13071-025-06956-z)
Supplement: Supplementary file 1 — Supplementary Material 1. [file 13071_2025_6956_MOESM1_ESM.docx]

**
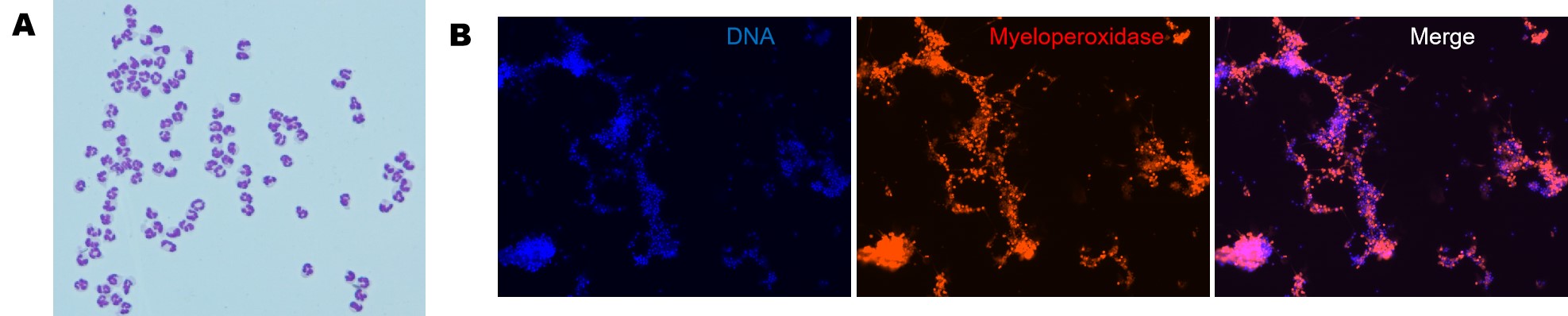
**

**Additional file: Fig. S1. Purity and immune function of the isolated neutrophils.** (A) Purified neutrophils were stained with Giemsa and observed under a microscope at 100× magnification. (B) Neutrophils stimulated with LPS (1 μg/mL) were assessed for NETs release using immunofluorescence microscopy. Hoechst 33342 was used to label DNA (blue), while Cy3 (red) was used to mark myeloperoxidase (40×). LPS, lipopolysaccharide; NETs, neutrophil extracellular traps

**
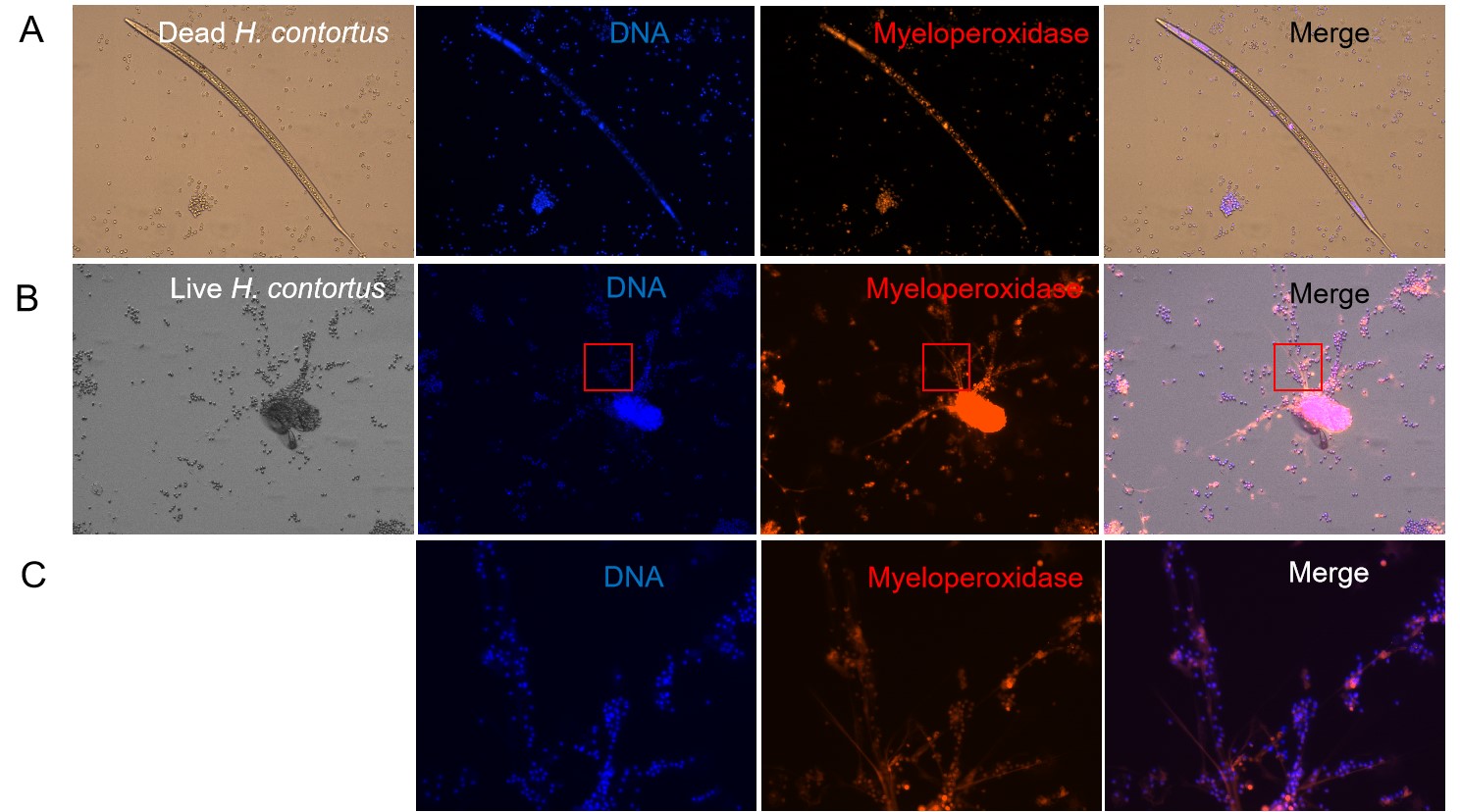
**

**Additional file: Fig. S2. HcL3 triggers the formation of NETs.** Hoechst 33342 was used to label DNA (blue), while Cy3 (red) was used to mark myeloperoxidase. (A) Neutrophils co-incubated with dead HcL3 (live HcL3 heat inactivated at 100℃ for 10min) (20×). (B) Neutrophils co-incubated with live HcL3 (20×). (C) Enlarge the area within the red box in (B) (40×). HcL3, *Haemonchus contortus* third-stage larvae


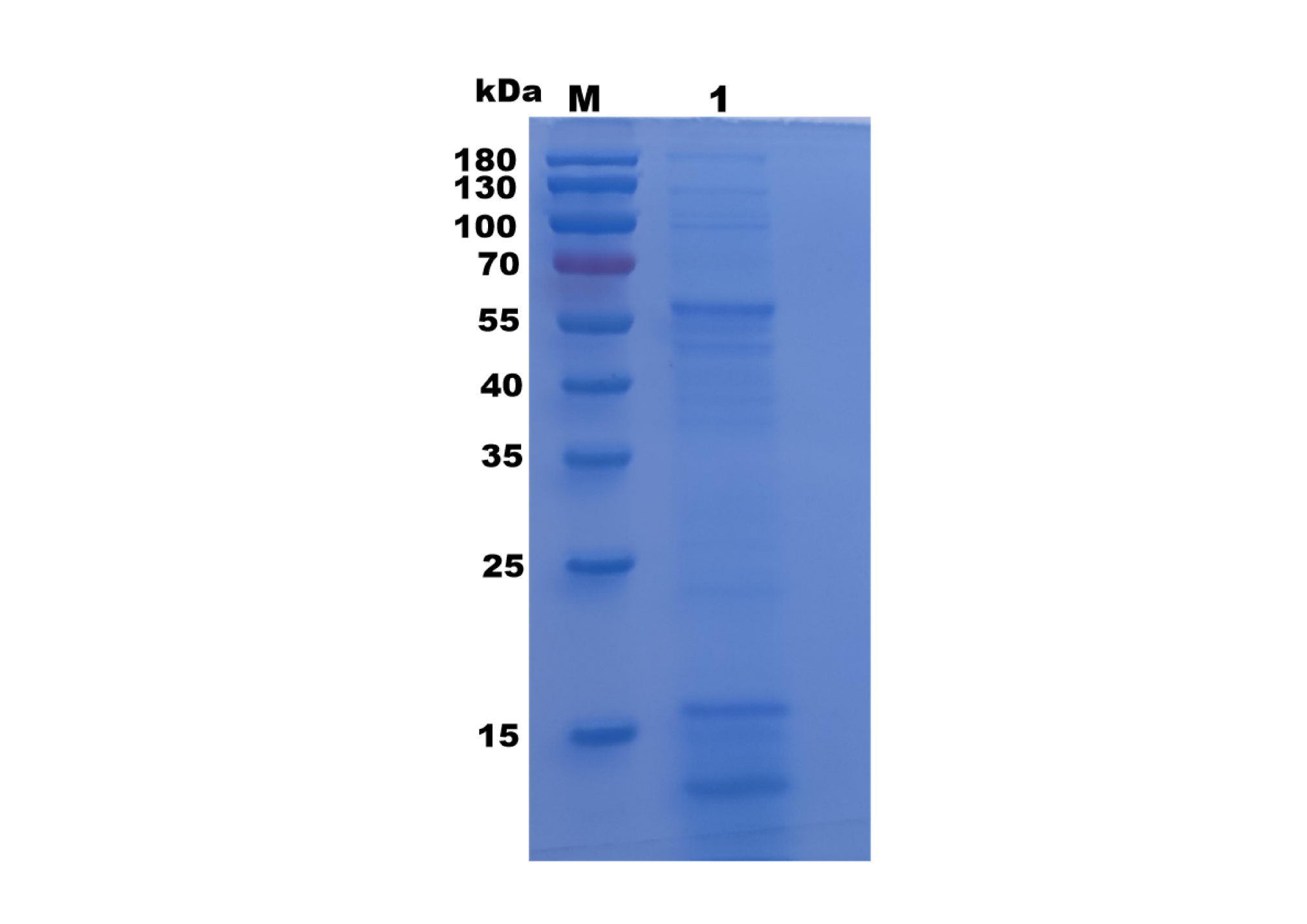


**Additional file: Fig. S3.** **SDS-PAGE of *Haemonchus contortus* excretory-secretory proteins.** Lane M: Standard protein molecular weight Marker; Lane 1: *H. contortus* excretory-secretory proteins with molecular weights ranging from 10 to 180 kDa. SDS-PAGE, sodium dodecyl sulphate–polyacrylamide gel electrophoresis; kDa, kilodalton
